# Fabrication of ILs-Assisted AgTaO_3_ Nanoparticles for the Water Splitting Reaction: The Effect of ILs on Morphology and Photoactivity

**DOI:** 10.3390/ma13184055

**Published:** 2020-09-12

**Authors:** Julia Zwara, Anna Pancielejko, Marta Paszkiewicz-Gawron, Justyna Łuczak, Magdalena Miodyńska, Wojciech Lisowski, Adriana Zaleska-Medynska, Ewelina Grabowska-Musiał

**Affiliations:** 1 Department of Environmental Technology, Faculty of Chemistry, University of Gdansk, 80-308 Gdansk, Poland; julia.zwara@phdstud.ug.edu.pl (J.Z.); m.paszkiewicz-gawron@ug.edu.pl (M.P.-G.); magdalena.miodynska@phdstud.ug.edu.pl (M.M.); adriana.zaleska-medynska@ug.edu.pl (A.Z.-M.); 2 Department of Process Engineering and Chemical Technology, Faculty of Chemistry, Gdansk University of Technology, 80-233 Gdansk, Poland; anna.pancielejko@pg.edu.pl (A.P.); justyna.luczak@pg.edu.pl (J.Ł.); 3 Institute of Physical Chemistry, Polish Academy of Science, 01-244 Warsaw, Poland; wlisowski@ichf.edu.pl

**Keywords:** ionic liquids, perovskite, hydrogen production/evolution, photocatalysis, water splitting

## Abstract

The design of an active, stable and efficient photocatalyst that is able to be used for hydrogen production is of great interest nowadays. Therefore, four methods of AgTaO_3_ perovskite synthesis, such as hydrothermal, solvothermal, sol-gel and solid state reactions, were proposed in this study to identify the one with the highest hydrogen generation efficiency by the water splitting reaction. The comprehensive results clearly show that the solid state reaction (SSR) led to the obtainment of a sample with an almost seven times higher photocatalytic activity than the other methods. Furthermore, four ionic liquids, all possessing nitrogen in the form of organic cations (two imidazoliums with different anions, ammonium and tetrazolium), were used for the first time to prepare composites consisting of AgTaO_3_ modified with IL and Pt, simultaneously. The effect of the ionic liquids (ILs) and Pt nanoparticles’ presence on the structure, morphology, optical properties, elemental composition and the effectiveness of the hydrogen generation was investigated and discussed. The morphology investigation revealed that the AgTaO_3_ photocatalysts with the application of [OMIM]-cation based ILs created smaller granules (<500 nm), whereas [TBA] [Cl] and [TPTZ] [Cl] ILs caused the formation of larger particles (up to 2 μm). We found that various ILs used for the synthesis did not improve the photocatalytic activity of the obtained samples in comparison with pristine AgTaO_3_. It was detected that the compound with the highest ability for hydrogen generation under UV-Vis irradiation was the AgTaO_3__0.2% Pt (248.5 μmol∙g^−1^), having an almost 13 times higher efficiency in comparison with the non-modified pristine sample. It is evidenced that the enhanced photocatalytic activity of modified composites originated mainly from the presence of the platinum particles. The mechanism of photocatalytic H_2_ production under UV-Vis light irradiation in the presence of an AgTaO_3__IL_Pt composite in the water splitting reaction was also proposed.

## 1. Introduction

All across the world, people are faced everyday with many forms of environmental pollution, such as: water, air and land pollution. These environmental problems affect every human, animal and plant [1,2,3,4]. The best solution would be to reduce or even remove the input of pollutants; however, this is impossible. Another very important problem that the world has to face today is the demand for energy [5,6,7]. Therefore, alternative solutions aimed at removing harmful substances from the air, water and soil, as well as ways for the acquisition of clean energy are being searched for [8,9,10,11]. One such solution is the application of photoactive material able to remove pollution and/or generate hydrogen in the presence of light with specific radiation. A challenge in the field of heterogeneous photocatalysis is to develop a new type of photoactive materials activated by low-powered and low-cost irradiation sources (also sunlight) [12,13,14]. Currently, hydrogen is mainly produced from carbon monoxide and natural gas (from fossil fuels) through a steam reforming reaction [15]. However, the risk of fossil fuel depletion, as well as the serious environmental problems associated with CO_2_ generation, has forced researchers to look for alternative solutions [16]. Recently, great interest has been focused on hydrogen production with a photocatalytic water-splitting reaction in the presence of semiconductor nanoparticles and UV-Vis or solar irradiation [17,18,19,20,21,22]. The basic requirements for developing photocatalysts for overall water splitting are: (i) sustainable conduction band (CB) and valence band (VB) edge potentials for overall water splitting, (ii) band-gap energy lower than 3 eV for visible-light harvesting, and (iii) photostability in time [23,24].

In this regard, the wide-bandgap semiconductors with d0 and d10 configuration such as Ti^4+^, Nb^5+^ and Ta^5+^ are used as photocatalysts for the degradation of pollutants and for hydrogen generation in the reaction of water-splitting [25,26]. Until now, the most commonly used materials were titanates (Ti 3d) because of their high ability to reduce water for H_2_ production. However, it was found that tantalate photocatalysts could be a better candidate since (i) the Ta^5+^ ion possesses higher reduction potential for hydrogen generation than most of the studied d0 elements [27] and (ii) the bond angle of Ta-O-Ta is close to 180 degrees, providing a high degree of delocalization and excellent mobility [28]. One of the very promising wide-bandgap semiconductors among tantaletes such as LiTaO_3_ [29], NaTaO_3_ [30], KTaO_3_ [31], CsTaO_3_ [32] is silver tantalate (AgTaO_3_) with a perovskite structure. However, to date, the practical applications of AgTaO_3_ are limited. It was reported that the AgTaO_3_ band gap of about 3.4 eV determined the ability to absorb only UV irradiation [33]. An additional problem is the low quantum yield and high recombination rate of the photogenerated charge carriers [34]. The density functional theory (DFT) calculations demonstrated that appropriate N/F co-doping could narrow the band gap of AgTaO_3_ to 2.9 eV while increasing the charge carrier mobility and the reductive strength towards hydrogen production [34]. Therefore, the following methods for increasing the photocatalytic performance of AgTaO_3_ were investigated: (i) co-doping [34], (ii) application of semiconductor composites [35] and (iii) modification with noble metals [36]. Among them, enhanced photocatalytic activity for hydrogen generation by the use of a co-catalyst was the most frequently investigated. As a result of the synergy of the interaction between the photocatalyst and the co-catalyst, effective separation of the photogenerated charge carriers occurs, due to changes in the semiconductor electronic structure, such as the band gap width or the position of the valence and the conduction bands. Recently, platinum was found to be the most effective co-catalyst owing to its largest work function and lowest overpotential for H_2_ evolution [37]. However, up to now, the research has focused mainly on the use of Ag nanoparticles on the AgTaO_3_ surface to enhance photocatalytic activity for hydrogen generation. For instance, Ag nanoparticles deposited on the AgTaO_3_ surface by means of a simple one-step chemical reduction treatment using ethylene glycol as a reducing agent allowed the preparation of the photocatalyst with a four-time increment for hydrogen production [38]. Yu et al. described the growth of Ag nanoparticles onto a AgTaO_3_/SrTiO_3_ solid solution using an in situ exolution procedure with ethylene glycol [39]. The presence of a co-catalyst led to the enhancement of hydrogen generation by nearly 45% due to the localized surface plasmon effect. Photodeposition of Ag and Cu nanoparticles onto AgTaO_3_ perovskite for improved photocatalytic hydrogen evolution was also reported [36]. According to our knowledge, there are no reports regarding the use of AgTaO_3_ decoration using Pt nanoparticles for hydrogen evolution.

Another, actually surprising, way to improve the wide-bandgap semiconductor photoactivity under visible light is the use of ionic liquids (ILs) for photocatalyst preparation [40]. Semiconductor modification with ionic liquids is a new, effective approach, but the mechanism of their action is not yet fully explained. It is known that ionic liquids form a protective layer on the semiconductor particle surface, thus electrosteric solvation and viscous stabilization of the growing particles occurs [41]. The presence of ionic liquids in the reaction system can also promote the formation of oxygen vacancies, which can be a source of the electronic charge required for O_2_ adsorption and intermediate energy level [42]. Additionally, ILs as organic compounds possess HOMO-LUMO levels. In this regard, between TiO_2_, being an n-type semiconductor, and the halogen anion of an IL (where the HOMO orbital is located), new energy levels can be formed [43]. The literature reports that ILs introduced during the preparation of a broadband photocatalyst may increase its activity under visible light due to: (i) doping of non-metal elements (e.g., N, B, F) derived from the IL structure, inducing a narrower band gap and improving the separation efficiency of the photogenerated electron/hole pairs [44]; (ii) it favoring oxygen vacancies [42]; (iii)surface complex charge transfer [45] and (iv) it affecting transport of photogenerated charges [46]. As far as we know, no one has investigated the photocatalytic activity of IL_AgTaO_3_ loaded with Pt nanoparticles towards hydrogen generation.

**Figure 1:**
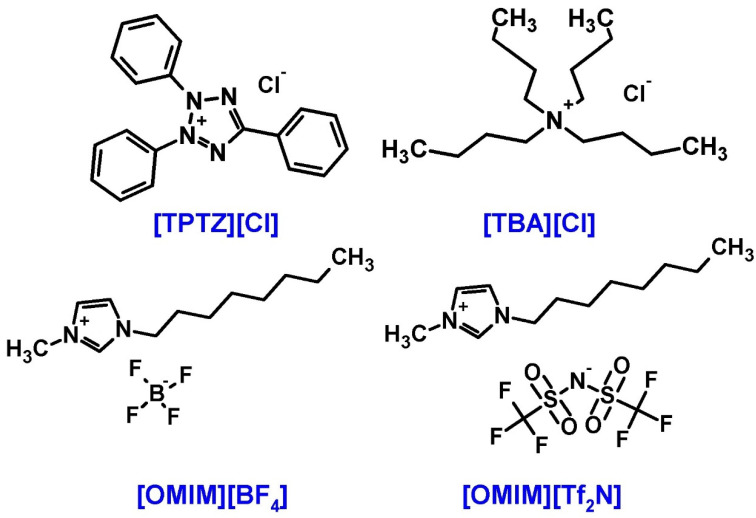
Structure of the ionic liquids (ILs) applied during the synthesis of AgTaO_3_: 2,3,5-triphenyltetrazolium chloride [TPTZ] [Cl], tetrabutylammonium chloride [TBA] [Cl], 1-methyl-3-octylimidazolium tetrafluoroborate [OMIM] [BF_4_], 1-methyl-3-octylimidazolium bis(trifluoromethylsulfonyl)imide [OMIM] [Tf_2_N].

Although AgTaO_3_ has been studied for different applications, no one has reported the comparisons of four different synthesis methods, such as solvothermal (SS), sol-gel (SG), hydrothermal (HS) and solid-state reactions (SSR), to synthesize the photocatalyst with a desirable structure, morphology and enhanced photocatalytic activity using the water splitting reaction. Moreover, for the first time, the effect of ILs differing in structure, namely 2,3,5-triphenyltetrazolium chloride [TPTZ] [Cl], tetrabutylammonium chloride [TBA] [Cl], 1-methyl-3-octylimidazolium tetrafluoroborate [OMIM] [BF_4_], 1-methyl-3-octylimidazolium bis (trifluoromethylsulfonyl) imide [OMIM] [Tf_2_N] (structural formulas shown in Figure 1) and Pt nanoparticles’ presence on the morphology and photoactivity of AgTaO_3_ has been investigated.

## 2. Materials and Methods

### 2.1. Materials

Silver nitrate (POCh S.A., Gliwice, Poland), sodium hydroxide (Chempur, Piekary Slaskie, Poland), tantalum oxide (99%, Sigma Aldrich, Darmstadt, Germany) were used for synthesis of AgTaO_3_ semiconductors. Ionic liquid, 2,3,5-triphenyltetrazolium chloride [TPTZ] [Cl] (>98%) and tetrabutylammonium chloride [TBA] [Cl] (>97%) were purchased from Sigma Aldrich (Darmstadt, Germany). 1-methyl-3-octylimidazolium tetrafluoroborate [OMIM] [BF_4_] and 1-methyl-3-octylimidazolium bis(trifluoromethylsulfonyl)imide [OMIM][Tf_2_N] with a purity of >99% from Iolitec (Heilbronn, Germany).

### 2.2. Preparation of AgTaO_3_

At first, we decided to use four different methods, namely solvothermal, sol-gel, hydrothermal and solid state reactions to prepare the perovskite. The applied synthesis procedures were as follows:

- Preparation of AgTaO_3_ by the solvothermal method. The AgTaO_3_ powder was obtained as follows: 0.95 g AgNO_3_ was dissolved in 120 mL of ethylene glycol and then 2.02 g TaCl_5_ was added. The solution was stirred for 15 min. The resulting mixture was transferred into a Teflon-lined stainless steel autoclave (LabPartner, Warsaw, Poland) and treated at 180 °C for 24 h. After cooling to room temperature, the obtained precipitate was separated by centrifugation, washed several times with deionized water, dried overnight at 60 °C and finally calcined at 800 °C for 4 h.
- Preparation of AgTaO_3_ by the sol-gel method. In the first step 2 g of AgNO_3_ was dissolved in 50 mL of deionized water and 4.19 g of TaCl_5_ was added. The mixture was kept under constant stirring conditions and 20 mL NH_4_OH was added dropwise to the above mixture. After stirring for 1 h, the resulting dark precipitate was separated by centrifugation, washed several times with deionized water and then dried at 60 °C until the liquid had completely evaporated. The obtained powder was further annealed at 800 °C for 2 h.
- Preparation of AgTaO_3_ by the hydrothermal method. In the hydrothermal route, Ag_2_O was first obtained. As in typical synthesis, NaOH (0.1 M) was slowly added under stirring conditions to AgNO_3_ (0.1 M). Then, the brown precipitation was collected and washed with deionized water several times, and dried overnight at 60 °C. The as-prepared Ag_2_O powder was mixed with Ta_2_O_5_, NH_4_HF_2_, H_2_O and H_2_O_2_. The solution was mixed for 10 min, transferred into a Teflon-lined stainless steel autoclave and treated at 180 °C for 24 h. After cooling to room temperature, the obtained precipitate was separated by centrifugation, washed several times with deionized water and dried overnight at 60 °C.
- Preparation of AgTaO_3_ by the solid state reaction. The precursor, Ag_2_O, was first obtained as described above. The as-prepared Ag_2_O was mixed with Ta_2_O_5_ in a stoichiometric ratio in the presence of Ag_2_O and ground by hand in an agate mortar. It is known that silver-based materials suffer a loss of silver at high calcination temperature. Therefore, to overcome this drawback, 3.0 wt% of Ag_2_O was added in excess to maintain the required stoichiometry [33,47]. The mixture was calcinated in air at 900 °C for 24 h, with a heating rate of 1 °C·min^−1^. After this process, the sample was naturally cooled down in a furnace to the ambient temperature.

### 2.3. Modification of AgTaO_3_ with IL and Co-Catalyst Pt by Using the Photodeposition Method

The IL-modified AgTaO_3_ powders were successfully prepared via a solid state reaction by homogenization Ag_2_O, Ta_2_O_5_ and IL in mortar (molar ratio of Ag_2_O to IL was constant and equaled 1:2) and calcinated at 900 °C for 24 h with a heating rate of 1 °C·min^−1^ (see Preparation of AgTaO_3_ by Solid State Reaction).

A suspension containing AgTaO_3_ or AgTaO_3__IL (2 g), 70 mL of ethanol solution and the platinum precursor K_2_PtCl_4_ (0.2 wt% of Pt) was placed in a quartz reactor and sonicated for 10 min. Then, the solution was degassed with nitrogen (8 dm^3^·h^−1^) and stirred in the dark for 30 min. The as-prepared suspension was irradiated with an Xe lamp (250 W, Heraeus Noblelight GmbH, Cambridge, UK) used as an irradiation source of UV for 1 h. The obtained samples were separated by centrifugation, sequentially rinsed with deionized water, and dried at 60 °C for 12 h. The specific concentration of platinum in the suspension was selected based on our previous research [48].

### 2.4. Characterization of Materials

The crystal structure of the samples obtained was characterized by the X-ray powder diffraction method (XRD, Rigaku MiniFlex 600, Rigaku, The Woodlands, TX, USA) measured in the 2θ range of 20–80° with the target Cu Kα irradiation. The mean crystallite size from the Scherrer equation was also estimated. The shape and size of the particles were observed by scanning electron microscopy (SEM, JEOL JSM-7610F, Jeol Ltd., Tokyo, Japan). The surface content of the samples was determined by X-ray photoelectron spectroscopy (XPS, PHI 5000 VersaProbe^TM^, ULVAC-PHI, Chigasaki, Japan) with a source of monochromatic Al Kα irradiation (hν = 1486.6 eV). High-resolution spectra (HR-XPS) were measured using a hemispherical analyzer (transition energy 23.5 eV, energy step size 0.1 eV). The recorded C1s spectrum of carbon was used as reference for binding energy (284.8 eV). The BET (Gemini V (model 2365)) surface area was determined by a multipoint method with the use of adsorption data in the relative pressure (P/P0) range of 0.05–0.3 after degassing the samples at 200 °C. The diffuse reflectance spectra (UV-Vis) were recorded with a spectrophotometer (Evolution 220, Thermo Fisher Scientific, Waltham, MA, USA) in the scanning range of 200–900 nm. The spectrophotometer was equipped with an integrating sphere accessory for diffuse reflection with the baseline performed using barium sulphate. Fourier transformed infrared spectra (FTIR) were obtained with a Nicolet iS10 FTIR spectrometer in a scanning range of 500–4000 cm^−1^ with a resolution of 4 cm^−1^. Before analysis, the samples were prepared by diluting in KBr 5% of the photocatalysts. Raman spectra were recorded a DXR Smart Raman on spectrometer. A laser emitting irradiation with a wavelength of 532 nm was used as the excitation source.

### 2.5. Measurements of Photocatalytic Activity in Water-Splitting Reaction

The photocatalytic hydrogen evolution experiments were carried out in a tightly closed cylindrical quartz reactor. In a typical experiment, the photocatalyst (0.1 g) was dispersed with continuous stirring (700 rpm) in an aqueous methanol solution (80 mL, C = 10%), which was used as a sacrificial reagent for holes (h^+^). The process was carried out at a constant temperature of 10 °C set by a thermostatically controlled water bath. The space above the suspension was purged with nitrogen for 30 min to remove residual oxygen, and then the system was irradiated with a 1000 W Xe lamp (Oriel Instruments, Stratford, CT, USA) which emitted UV-Vis irradiation. The evolved gas (200 μL) was collected through the septum at regular time intervals every 60 min using a gas-tight syringe. The total exposure time of the sample was 240 min (in the case of testing, the exposure time of the most photoactive composite was 20 h). The amount of hydrogen generated in the tested samples was analyzed using a gas chromatograph (Trace 1300, Thermo Fisher Scientific, Waltham, MA, USA) equipped with a thermal conductivity detector (TCD) with N_2_ as the carrier gas and with a column (HayeSep Q (80/100)). Hydrogen generation was determined by a blank test in the absence of a photocatalyst, where evolution of H_2_ was not observed. The specific conditions for conducting the hydrogen generation process (type and concentration of sacrificial agent (10% methanol) as well as the amount of the photocatalyst (0.1 g)) were established based on our previous research [48]. Additionally, the measurement with a glass filter (GG420, Optel, Opole, Poland) cutting off wavelengths shorter than 420 nm revealed no hydrogen generation.

## 3. Results and Discussion

**Figure 2:**
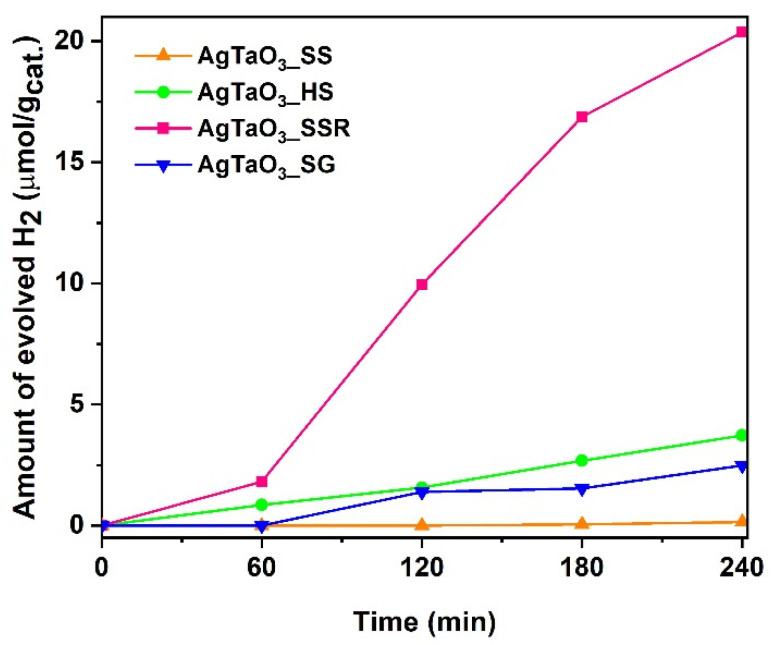
Efficiency of H_2_ generation determined for AgTaO_3_ prepared via different methods, namely solvothermal synthesis (AgTaO_3__SS), hydrothermal synthesis (AgTaO_3__HS), solid state reaction (AgTaO_3__SSR) and sol-gel (AgTaO_3__SG).

Firstly, the preparation routes of the AgTaO_3_ synthesis was taken into consideration. Four different methods were applied, and based on the obtained results, including crystallite size (Table S1) and hydrogen evolution in the water splitting reaction (see Table S1, Figure 2), it was concluded that the technique which led to the obtainment of AgTaO_3_ with the smallest crystallite size, and thus with the highest ability to generate hydrogen, was SSR in comparison with the other methods—SS, HS, SSR and SG. Therefore, we decided to select SSR for the preparation of the IL-modified samples followed by surface decoration with Pt particles using the photodeposition method.

At the next step, the effect of the ILs’ structure on the crystalline structure, morphology and photoactivity of AgTaO_3_ was considered, and four different ILs were chosen, namely [TPTZ][Cl], [TBA][Cl] and [OMIM][BF_4_], [OMIM][Tf_2_N]. The molar ratio of ILs to Ag_2_O was constant and equaled 1:2. Based on our previous research on the perovskite materials, we selected the amount of platinum precursor at the level of 0.2 wt% [48].

### 3.1. Morphology

**Figure 3:**
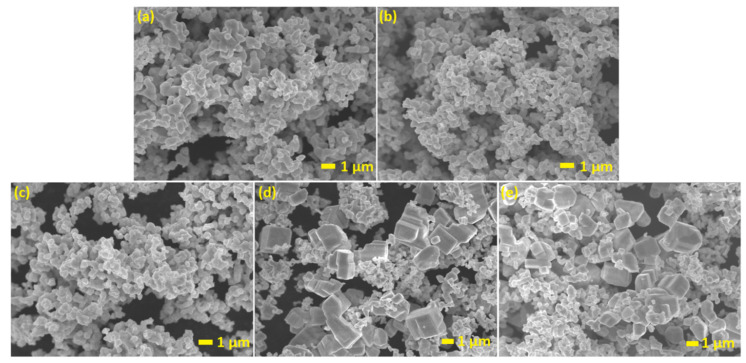
SEM images of: (**a**) pristine AgTaO_3_, (**b**) AgTaO_3__[OMIM][BF_4_]_0.2% Pt, (**c**) AgTaO_3__[OMIM][Tf_2_N]_0.2% Pt, (**d**) AgTaO_3__[TBA][Cl]_0.2% Pt, (**e**) AgTaO_3__[TPTZ][Cl]_0.2% Pt.

The microstructures of as-prepared powders were inspected under electron microscopy conditions. Typical SEM images of the following samples (a) pristine AgTaO_3_, (b) AgTaO_3__[OMIM][BF_4_]_0.2% Pt, (c) AgTaO_3__[OMIM][Tf_2_N]_0.2% Pt, (d) AgTaO_3__[TBA][Cl]_0.2% Pt, (e) AgTaO_3__[TPTZ][Cl]_0.2% Pt are shown in Figure 3. The obtained powder samples consisted of irregular particles with a smooth surface where the size and shape depended on the ionic liquids. It was found that pristine AgTaO_3_ and that modified with both [OMIM][BF_4_] and [OMIN][[Tf_2_N] ionic liquids and Pt nanoparticles were composed of granules smaller than 500 nm (Figure 3a–c). The particles size increased up to 2 µm when AgTaO_3_ was modified by [TBA][Cl] and [TPTZ][Cl] (Figure 3d,e). What is more, the formation of asymmetrical cubes for those samples was observed.

### 3.2. The XRD and BET Analyses

**Figure 4:**
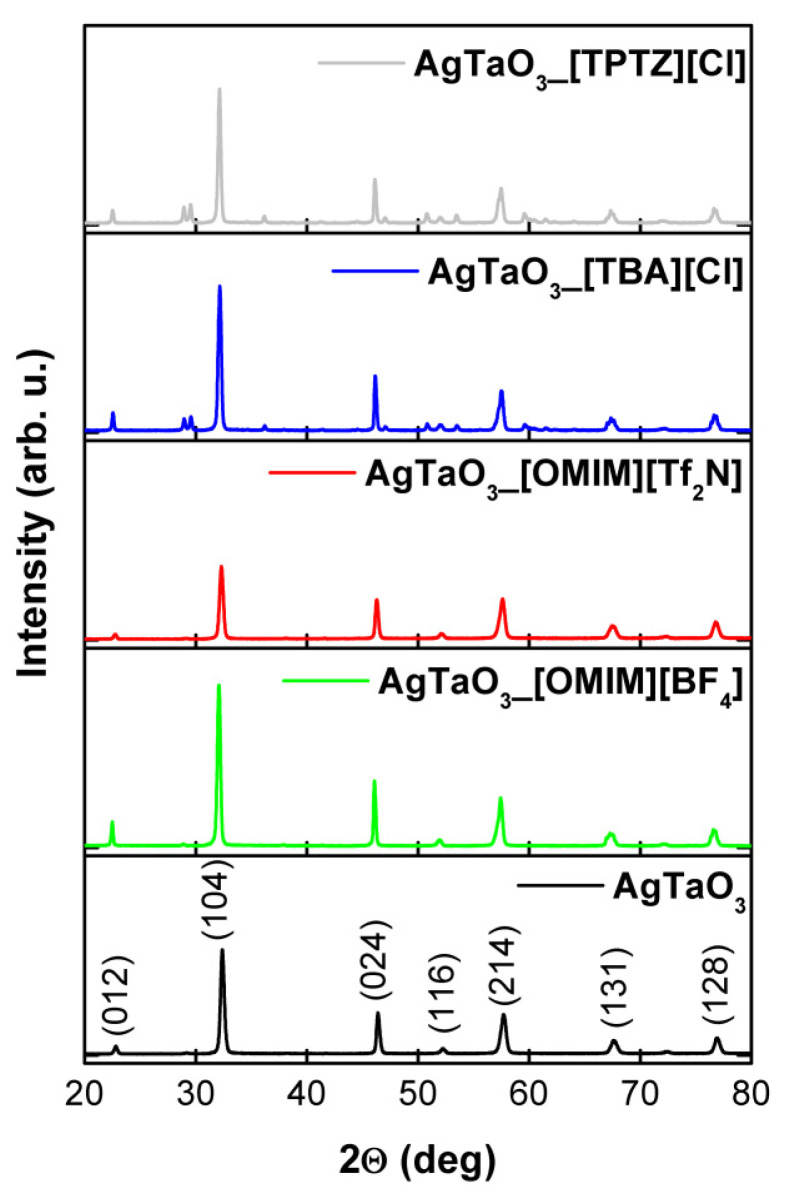
XRD patterns of pristine and IL-modified AgTaO_3_ samples.

The XRD patterns of the as-prepared samples are shown in Figure 4. The peaks near 22.8°, 32.6°, 46.3°, 52.2°, 57.7°, 72.4° and 76.9° corresponded to a pure phase of AgTaO_3_. Calcination of these samples at 900 °C for 24 h led to the formation of AgTaO_3_ nanoparticles with a rhombohedral perovskite type structure with R3c space group. The refined lattice parameters a, b and c, unit cell volume, and average crystallite size are gathered in Table 1. The addition of an IL to the reaction environment caused changes in the intensity of the (104) peak in comparison with the pristine sample. The samples prepared in the presence of [TPTZ] [Cl] and [TBA] [Cl] possessed additional peaks which could originate from the ILs residual impurities. Decoration with Pt nanoparticles did not have any influence on the peak position, which indicated that Pt was deposited on the surface instead of being inserted in the crystal lattice of AgTaO_3_. Furthermore, no peaks derived from Pt were observed. This is probably due to their high dispersion and low content on the AgTaO_3_ photocatalyst. The average crystallite size was estimated based on the Scherer equation. The discrepancies in the crystallite sizes of the modified samples in comparison with the reference AgTaO_3_ are thought to originate from the presence of different ILs structures and the results were collected in Table 1. As can be observed, especially in the case of [TPTZ] [Cl], the increase in crystallite size was the largest, and changed from 215.4 to 294.5 Å, for AgTaO_3_ and AgTaO_3__[TPTZ] [Cl], respectively. Moreover, it was observed that for AgTaO_3__0.2% Pt, and AgTaO_3__[OMIM] [Tf_2_N]_0.2% Pt, the average crystallize size increased, from 215.4 to 259.4 Å and from 218.2 to 262.8 Å, respectively. However, for the rest of the samples modified by ILs and Pt, a decrease in average crystallite size was observed. Regarding the specific surface area, the results determined for AgTaO_3__[OMIM] [BF_4_] and AgTaO_3__[OMIM] [Tf_2_N] were around 0.87 and 1.12 m^2^·g^−1^, respectively, whereas the area of the pristine AgTaO_3_ sample equaled 0.92 m^2^·g^−1^ (as presented in Table 1). When the [TBA] [Cl] and [TPTZ] [Cl] were added into the reaction environment, the specific surface area of the modified perovskites equaled 0.56 m^2^·g^−1^ for AgTaO_3__[TBA] [Cl] and 0.7256 m^2^·g^−1^ for AgTaO_3__[TPTZ] [Cl]. Moreover, Pt surface deposition resulted in an increase in the specific surface area for AgTaO_3__0.2% Pt (1.1408 m^2^∙g^−1^), AgTaO_3__[OMIM] [BF4]_0.2% Pt (1.0362 m^2^∙g^−1^) and AgTaO_3__[TBA] [Cl]_0.2% Pt (0.7214 m^2^∙g^−1^), whereas for the rest, BET surface area decreased (see Table 1).

### 3.3. The XPS Analyses

**Figure 5:**
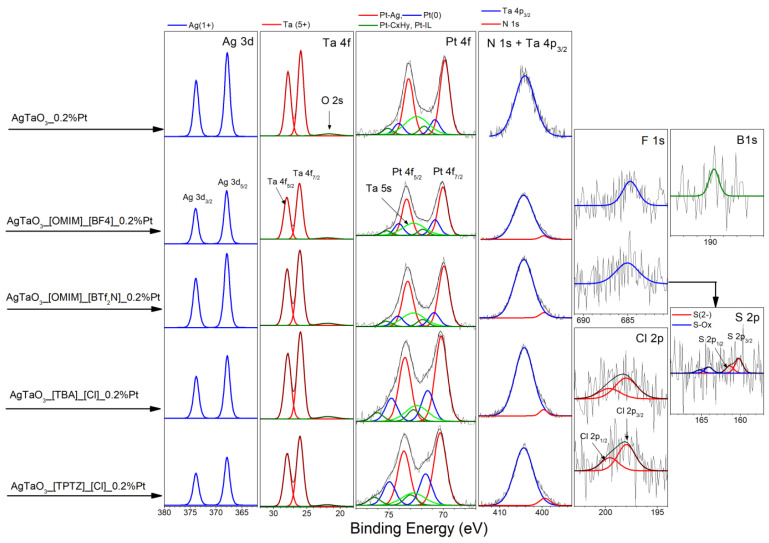
The high resolution XPS spectra of elements detected in AgTaO_3__0.2% Pt and IL-modified AgTaO_3__0.2% Pt composites.

The elemental composition in the surface region of pristine AgTaO_3_ and the IL-modified AgTaO_3__0.2% Pt composites was determined by XPS and collected in Table 2. The HR spectra of Ag 3d, Ta 4f and Pt 4f, presented in Figure 5, identify well Ag, Ta and Pt as main elements of these samples [49]. Detection of fluorine (F1s spectrum) and boron (B1s spectrum) in AgTaO_3__[OMIM][BF_4_] and fluorine and sulphur (S2p spectrum) in AgTaO_3__[OMIM] [Tf_2_N] evidences the successful modification of AgTaO_3__Pt samples by [OMIM] [BF_4_] and [OMIM] [Tf_2_N] ionic liquids, respectively. The Cl 2p spectra recorded on AgTaO_3__[TPTZ] [Cl] and AgTaO_3__[TBA] [Cl] samples confirm the successful modification of AgTaO_3_ with [TPTZ] [Cl] and [TBA] [Cl] IL, respectively. However, nitrogen, originated from all IL dopants, was detected in the BE region of N 1s overlapped by intensive Ta 4p_3/2_ signals. Thus, the deconvolution of these complex spectra was necessary to evaluate the nitrogen content in all samples (Table 2). Similarly, the Pt 4f spectra were partially overlapped by the Ta 5s signals. However, after deconvolution, three Pt states were identified, represented by Pt 4f_7/2_ signals, located at BE of 69.9–70.2, 70.8–71.4 and 71.8–72.7 eV (see Pt 4f_7/2_ fractions named as Pt1, Pt2 and Pt3, respectively in Table 2). The first Pt state (Pt1) is addressed to Pt-Ag bonds formed as a result of the Pt interaction with AgTaO_3_ [49], the second (Pt2) can be attributed to Pt(0) and Pt-CO adsorbate and the last one (Pt3) we assign to Pt bound formed by CxHy and IL surface species interacting with AgTaO_3_ [49]. The Pt1 state is a dominant fraction of Pt compounds in the surface region of all samples. It is interesting to note that both chloride composites, namely AgTaO_3__[TBA] [Cl] and AgTaO_3__[TPTZ] [Cl], exhibit a larger platinum content than the other samples (Table 2), which suggests the segregation of Pt to the surface region of these samples. This supposition is supported by the Pt/Ag ratios of both samples, being about two times higher than the other ones (Table 2). The larger surface amount of Pt in these samples is accompanied by a larger amount of carbon species (see C/Ag ratios of both samples in Table 2), which indicates a larger concentration of IL at the surface. The increased amount of IL adsorbate is also detectable in the Pt 4f spectra of both samples. We observed a relative decrease in Pt1 and an increase in Pt3 fractions contributing to the Pt 4f spectra (Table 2).

### 3.4. The FTIR and Raman Analyses of Lattice Vibration Modes

The FTIR and Raman analyses carried out confirmed the obtainment of the AgTaO_3_ structure. The FTIR spectra of pristine and IL-modified AgTaO_3_ are shown in Figure S1a. All samples exhibited similar spectral features, with the most characteristic aspect being the high-intensity infrared band around 414, 542 and 831 cm^−1^ and corresponding to the Ag–O bonds. The analysis revealed the presence of some characteristic peaks corresponding to ILs. For instance, for the samples modified with [TBA] [Cl] and [TPTZ] [Cl], the peaks indexed to the C–Cl bonds were observed at around 872 cm^−1^. In the spectrum of AgTaO_3__[TPTZ] [Cl], the band around 1025 cm^−1^ was additionally observed corresponding to the “in-plane” C-H bending. The bands in the region of 1320–1460 cm^−1^ and 1580–1620 cm^−1^ ascribed to the C–C stretch (in ring) vibrations were also found. For the sample AgTaO_3__[OMIM] [Tf_2_N], the peak positioned around 438 cm^−1^ can be ascribed to the stretching vibration modes of S–S. Similar results were obtained by Raman spectroscopy, as presented in Figure S1b. The specific vibration modes are located around 125, 425, 488 and 601 cm^−1^, indicating the presence of pure phase of AgTaO_3_. The measured frequencies of peak positions for the samples modified by different ILs and Pt did not vary between each other. The slight differences in the intensities detected for the modified samples in comparison with the reference sample might have resulted from the preparation method.

### 3.5. Optical Properties

The UV-Vis absorption spectra of the pristine and IL-modified AgTaO_3_ perovskite loaded with 0.2 wt% Pt are presented in Figure S2. It can be noted that all of the obtained samples absorbed radiation mainly in the UV-Vis region. The application of ILs did not practically influence the results. However, in the case of the IL-modified samples, the absorption intensity was higher when a co-catalyst was deposited. Furthermore, the absorption band related to AgTaO_3_ from 300 to 370 nm represented the co-catalyst-decorated samples. It was observed that the absorption band of the perovskite modified with ILs and the Pt particles in visible light increased in intensity, whereas the red shift was negligible. It might suggest that the ability to absorb the higher wavelength mainly came from Pt co-catalyst particles deposited on the surface of the AgTaO_3_. Platinum particles were not observed on the spectrum, probably as a result of the overlap with the absorption spectrum of AgTaO_3_. Similar results were observed for AgMO_3_ (M = V, Nb, Ta) perovskite materials. The absorption band related to AgTaO_3_ from 200 to 350 nm was found in regard to the co-catalyst surface-loaded samples [33].

AgTaO_3_ belongs to the type of semiconductors with an indirect band gap, therefore its width was determined on the basis of the tangent lines in the plots of the square root of the Kubelka–Munk function vs. photon energy, as shown in Figure S3. It has been reported that the valence band of AgTaO_3_ perovskites is generally composed by O 2p states, which can be hybridized with Ag 4d states [50]. The tangent lines, which are extrapolated to (hνα)^1/2^ = 0, indicate the band-gap of 3.36 eV for pristine AgTaO_3_ and for the IL-modified samples 3.26 eV for [TPTZ][Cl] and [TBA][Cl] ILs and 3.30 eV for [OMIM][BF_4_] and [OMIM][Tf_2_N] ILs, respectively. The variation in the ILs structure did not extend the absorption range into the visible light region. The band gap of the samples decorated with Pt changed in the case of the [OMIM]-based cations of ILs, and was 3.33 eV. Thus, the AgTaO_3_ samples can absorb light in a longer wavelength region up to visible light. The value determined for the pristine AgTaO_3_ perovskite was in accordance with the literature, where the band gap was around 3.4 eV [51,52].

### 3.6. Photocatalytic Activity in the Water-Splitting Reaction

**Figure 6:**
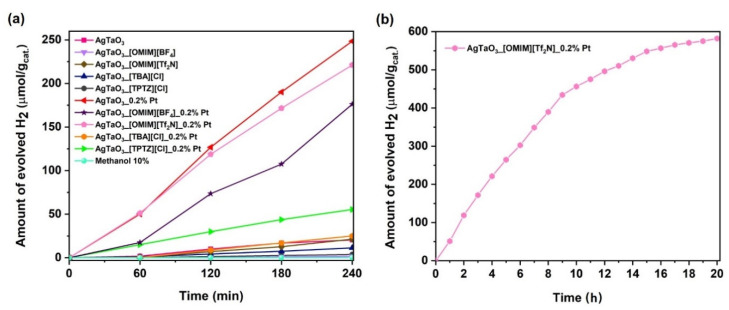
The amount of evolved H_2_ (**a**) under UV-Vis irradiation in the presence of the pristine and modified AgTaO_3_ photocatalyst, (**b**) under long-term UV-Vis irradiation in the presence of AgTaO_3__[OMIM][Tf_2_N]_0.2% Pt.

The photocatalytic activity of the obtained AgTaO_3_ perovskite materials for hydrogen production via photocatalytic water splitting, where methanol was used as a hole scavenger, was investigated and the results are presented in Figure 6 and Table 1. The procedure was developed based on our previous experimental studies in the following system: sacrificial reagent—methanol; concentration of methanol—10%; amount of the photocatalyst—0.1 g [48]. Before the main photocatalytic process, control tests were performed. The experiments in the presence of 10% methanol but without the addition of the photocatalyst revealed no H_2_ generation under UV-Vis irradiation. Moreover, under dark conditions, also no formation of hydrogen was detected.

The results indicate that the presence of the Pt particles on the AgTaO_3_ surface significantly improved the photocatalytic activity under UV-Vis irradiation. Firstly, it was found that pristine AgTaO_3_ exhibited photoactivity in hydrogen production even without any co-catalyst (20.4 μmol·g^−1^) hydrogen after 4 h of irradiation (see Figure 6a). This is because the photogenerated electrons could be transferred to Ag^+^ via the interface to accelerate the charge separation and thus influence the photocatalytic efficiency [53]. Hydrogen generation from pristine AgMO_3_ perovskite materials (M = V, Nb, Ta) was also observed by Moctezuma et al. [33]. As a result of the 3-h irradiation, they obtained 136 μmol g^−1^ of hydrogen in the presence of Na_2_SO_3_ 0.5 M as a sacrificial agent solution. Carrasco-Jaim et al. also received different hydrogen production efficiency (27 µmol after 3 h irradiation) [36]. Large differences in the efficiency of the conducted experiments could result from the different preparation route used to synthesize AgTaO_3_ perovskite. Secondly, we noticed that the ILs modification did not influence the enhancement of the photocatalytic activity of AgTaO_3_ composites compared to the reference sample. Moreover, the results show the opposite effect and the samples exhibited even lower photoactivity than the pristine AgTaO_3_ (see Table 1). Only application of [OMIM] [Tf_2_N] caused slightly higher H_2_ evolution (21.3 μmol g^−1^). In the next step, we analyzed the IL-modified samples with Pt loaded at the surfaces. The largest amount of H_2_ was achieved for AgTaO_3__0.2% Pt contributed to the highest H_2_ evolution rate, 248.5 μmol·g^−1^ after 240 min under UV-Vis irradiation (almost 13 times higher than for the pristine sample). In each case, the amount of H_2_ evaluated was slightly different and could depend directly on the structure and properties of the ILs. Lower values of H_2_ production were observed for AgTaO_3__[OMIM] [BF_4_]_0.2% Pt (176.2 μmol·g^−1^) and AgTaO_3__[OMIM] [Tf_2_N]_0.2% Pt (221.2 μmol·g^−1^) after 240 min under UV-Vis irradiation. For the samples of AgTaO_3__[TPTZ] [Cl]_0.2% Pt and AgTaO_3__[TBA] [Cl]_0.2% Pt generated almost 5 and 10 times lower amounts of H_2_, 55.4 and 25.1 μmol g^−1^, respectively. Additionally, no other gases were detected during the process. These results indicate the possible transfer of the excited electrons and photogenerated holes from the valence band AgTaO_3_ after the surface Pt deposition to the conduction band. According to the literature, the low Schottky barriers of metal semiconductor surfaces act as electron traps, facilitating electron-hole separation and catalyzing the proton reduction to H_2_ molecules and thus the enhancement of their photoactivity [54].

Catalyst stability was probed by a long-term (20 h) hydrogen evolution test in the presence of the sample which exhibited the highest photoactivity in hydrogen generation among all modified composites, AgTaO_3__[OMIM] [Tf_2_N]_0.2% Pt (Figure 6b). As a result, hydrogen was constantly produced in the presence of the composite AgTaO_3__[OMIM] [Tf_2_N]_0.2% Pt. The yield of hydrogen generation after 20 h of irradiation equaled 582.1 μmol g^−1^.

### 3.7. Discussion of Photocatalytic Mechanism

The AgTaO_3__0.2% Pt sample exhibited the highest hydrogen evolution under UV-Vis irradiation (248.5 μmol·g^−1^). The samples modified with different ILs, namely [OMIM][BF_4_], [OMIM][Tf_2_N], [TBA][Cl] and [TPTZ][Cl], followed by decoration with Pt particles showed lower photoactivity in the water splitting reaction under the same conditions. The present paper aims to call into question: why does the addition of ILs suppress hydrogen evolution? Did the improvement in photoactivity originate only from the interaction between AgTaO_3_ and platinum? What was the role of ILs in the production of H_2_?

To answer these questions, we need to carefully examine the above-mentioned results and the mechanisms of the photocatalytic reactions analyzed previously by our group. Based on the available literature and our own experience, it is commonly known that ILs play a significant role in the increase in photoactivity in the degradation of the aqueous phenol solution, MO and RhB solution, and photocatalytic hydrogen production [20,40,41,45,46,55,56]. Recently, we analyzed the effect of the various ILs used in the solvothermal reaction over TiO_2_ particles, and the results clearly show that the significant impact on the photocatalytic performance originated directly from interaction between photocatalyst particles and ILs [40,41,43,45]. The photoexcitation of the TiO_2_ samples modified with ILs occurred directly through the formation of a surface complex which resulted in the transfer of electrons from the LUMO orbit of the ionic liquid to the conductivity band TiO_2_. It was found that synthesis conditions of the solvothermal route allowed for the successful decomposition of the ILs, which resulted in the incorporation of nitrogen into the TiO_2_ structure and thereby significantly improved the photocatalytic activity under UV-Vis and Vis irradiation [41,43]. Qi et al. also investigated the effect of ILs on the photocatalytic activity of the TiO_2_ semiconductor. They realized that addition of an IL with a [Bmim]^+^ cation slightly enhanced the photocatalytic degradation rate of MO due to enhanced trapping and transfer of the photogenerated electrons. On the other hand, presence of an IL suppressed the degradation rate of RhB on the TiO_2_ surface by restricting the diffusion of positively charged holes to the TiO_2_/solution interface [46]. On the other hand, investigation of the hydrogen evolution using IL-modified SrTiO_3_ perovskite followed by surface photodeposition of Pt nanoclusters did not reveal a direct correlation between the increase in H_2_ evolution due to the presence of the IL [48]. Furthermore, we found that the enhancement of the photoactivity originated mainly from the Pt loaded on the photocatalyst surface, not from the presence of the IL. In this research, we suppose that the addition of ILs during the synthesis stage (the ILs were grated with Ag_2_O and Ta_2_O_5_ in the molar ratio 1:2 vs. Ag_2_O: IL) could have resulted in the formation of a monolayer which stuck to the powder and decomposed during calcination in the high temperature. The XPS analysis revealed the presence of the residual elements derived from the IL, namely fluorine and boron for AgTaO_3__[OMIM] [BF_4_], fluorine and sulphur for AgTaO_3__[OMIM][Tf_2_N] and chlorine for samples AgTaO_3__[TPTZ] [Cl] and AgTaO_3__[TBA] [Cl] (see Table 2 and Figure 5). Moreover, the FTIR measurements also confirmed the presence of bonds derived from liquids, such as C–Cl from [TBA] [Cl], C–Cl, CH and C–C (in ring) from [TPTZ] [Cl], as well as S–S bonds derived from [OMIM] [Tf_2_N] (Figure S2). Additionally, it was found that the presence of ILs mainly influenced the formation of nanoparticles which consisted of granules smaller than 500 nm, when [OMIM] [BF_4_] and [OMIM] [Tf_2_N] were used (similar morphological parameters as for pristine sample), and asymmetric cubes with a size of about 2 μm, for the [TBA] [Cl] and [TPTZ] [Cl] ionic liquids, respectively.

**Figure 7:**
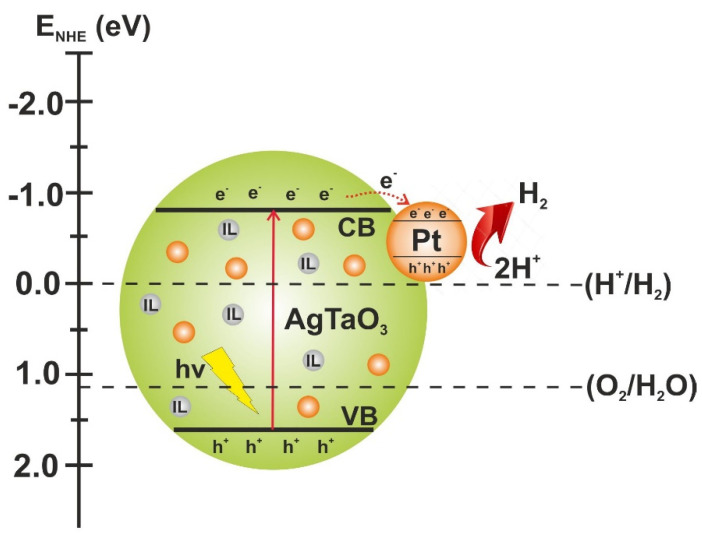
Proposed mechanism of photocatalytic H_2_ production under UV-Vis light irradiation in the presence of AgTaO_3__IL_Pt.

The water splitting measurements were performed firstly for the non-modified and IL-modified samples. As expected, without the co-catalyst, the samples showed very poor performance. Only the AgTaO_3__[OMIM] [Tf_2_N] showed slightly higher H_2_ evolution in comparison with the non-modified AgTaO_3_ sample (21.3 and 20.4 μmol·g^−1^, respectively), whereas the other samples exhibited much lower photoactivity. As a result of the photodeposition of the Pt nanoparticles, enhanced H_2_ generation in comparison with the non-modified pristine sample was found. Figure 7 shows the proposed mechanism of photocatalytic H_2_ production under UV-Vis light irradiation in the presence of AgTaO_3__IL_Pt. As a result of the absorption of UV-Vis irradiation, the photocatalyst was excited, generating pairs of charge carriers (e^−^-h^+^). High-energy electrons from the conduction band were transferred to Pt particles, where they participated in the reduction of water to molecular hydrogen. Aldoni et al. achieved the same effect [57]. According to the literature, it was found that the noble metal nanoparticles adsorbed on the surface of photocatalysts provided additional reaction sites and act as effective electron traps for photogenerated electrons due to the formation of the Schottky barrier at the metal-semiconductor contact point and promotion of the charge carriers separation [58]. The highest H_2_ production was observed for AgTaO_3__0.2% Pt (248.5 μmol·g^−1^), whereas all the modified samples with both an IL and Pt possessed lower photoactivity (see Table 2 and Figure 6a). Interestingly, the Pt content for the most active sample modified with both an IL and Pt (AgTaO_3__[OMIM] [Tf_2_N]_0.2% Pt) was the lowest, and amounted to 0.74 at %. Moreover, the composites which exhibited the lowest H_2_ evolution, AgTaO_3__[TBA] [Cl]_0.2% Pt and AgTaO_3__[TPTZ] [Cl]_0.2% Pt, revealed the highest Pt concentration, 1.20 and 1.28 at.%, respectively. In addition, the C content in those composites was the highest, 19.23 and 16.23 at.%, respectively. The C/Ag ratio for both of those samples was also the highest (see Table 2). It is evidenced that these two ILs could be adsorbed in a larger amount in the form of the residual elements derived from ILs at the composite surface, and suppressed the photoactivity in H_2_ generation. Therefore, even higher Pt content adsorbed on the surface of those composites did not enhance the photocatalytic efficiency.

Some literature reports associated higher H_2_ generation with larger specific surface area and crystallinity, which promotes more active sites for gas evolution, improving the transfer process of photogenerated charge pairs and thus enhancing the photocatalytic activity [59,60]. Based on the previously discussed experimental data, a direct relation between the increase in the BET surface area and the improvement of photocatalytic H_2_ production was observed. The AgTaO_3__0.2% Pt sample possessed the largest BET surface area and also the highest hydrogen evolution compared to the other samples, 1.1408 m^2^·g^−1^ and 248.5 μmol·g^−1^, respectively. Hence, the samples with the smallest surface area, AgTaO_3__[TPTZ][Cl]_0.2% Pt (0.7124 m^2^·g^−1^ ) and AgTaO_3__[TBA][Cl]_0.2% Pt (0.6989 m^2^·g^−1^) generated much lower amounts of H_2_ (55.4 and 25.1 μmol·g^−1^, respectively). Evidently, the addition of ILs to the reaction medium reduced the specific BET surface area and suppressed the photocatalytic activity.

## 4. Conclusions

Silver tantalate was successfully prepared via a solid-state reaction in the presence of four ILs differing in structure, namely, 2,3,5-triphenyltetrazolium chloride [TPTZ][Cl], tetrabutylammonium chloride [TBA][Cl], 1-methyl-3-octylimidazolium tetrafluoroborate [OMIM][BF_4_], 1-methyl-3-octylimidazolium bis(trifluoromethylsulfonyl)imide [OMIM][Tf_2_N], followed by surface platinum nanoparticle decoration using the photodeposition method. Morphology analysis revealed granules smaller than 500 nm in size when the samples were prepared in the presence of imidazolium ILs, and cubic shaped particles around 2 μm in size when ammonium and tetrazolium ILs were applied. We found that the various ILs used in the synthesis did not improve the photocatalytic activity of the obtained samples in comparison to pristine AgTaO_3_. The enhanced hydrogen generation came only from the presence of Pt nanoparticles on the photocatalyst’s surface, not from the IL modification. Despite the confirmed interactions between IL and AgTaO_3_ and its influence on the morphology, optical and photocatalytic properties, we suppose that ILs might block the sample surface and thus lower the photocatalytic activity. We assume that the reduced activity might result from the decomposition of the ionic liquid during the high calcination temperature, needed to obtain the final product AgTaO_3_. The samples which exhibited the highest C content (16.38 at.% for AgTaO_3__[TPTZ] [Cl] and 19.23 at.% for AgTaO_3__[TBA] [Cl]) and platinum content (1.28 at.% and 1.20 at.%, respectively) were characterized by the lowest H_2_ evolution. Moreover, the addition of IL to the reaction environment reduced the BET specific surface area and suppressed H_2_ generation. Among all the obtained samples, the compound with the highest ability to photocatalytically split water (248.5 μmol·g^−1^) was revealed to be AgTaO_3__0.2% Pt (almost 13 times higher efficiency in comparison with the non-modified pristine sample), while among the modified ILs, AgTaO_3__[OMIM] [Tf_2_N]_0.2% Pt (221.2 μmol·g^−1^). Interestingly, the Pt content for this sample was the lowest, and amounted to 0.74 at.%. Evidently, the enhanced H_2_ generation came from the presence of Pt nanoparticles on the composite’s surface and was suppressed due to use of ILs. The Pt nanoparticles promoted charge transfer from valence band to the AgTaO_3_ conduction band, and inhibited the recombination probability of the photogenerated electrons and holes, which was beneficial for the improvement in the photocatalytic activity of the modified samples. As a consequence, the ILs were responsible for the decrease in photocatalytic activity in the water splitting reaction. The MeOH electrolyte stabilized this photocatalyst during the extended photocatalytic process.

## Figures and Tables

**Table 1:** Parameters, average crystallite size and specific surface area.

Sample Label	a = b (A)	c (A)	V (A^3^)	Crystallite Size (A)	Specific Surface Area (m^2^·g^−1^)	Amount of H_2_ Evolved after 240 min (μmol g^−1^) under UV-Vis Irradiation
AgTaO_3_	5.4923	13.7718	359.78	215.4	0.9226	20.4
AgTaO_3__[OMIM][BF_4_]	5.8026	13.5746	366.07	269.5	0.8694	1.6
AgTaO_3__[OMIM][Tf_2_N]	5.5300	13.6315	361.01	218.2	1.1184	21.3
AgTaO_3__[TBA][Cl]	5.5415	13.7251	364.54	267.6	0.5617	11.3
AgTaO_3__[TPTZ][Cl]	5.5482	13.6898	359.78	294.5	0.7193	3.7
AgTaO_3__0.2% Pt	5.5667	13.7238	368.30	259.4	1.1408	248.5
AgTaO_3__[OMIM][BF_4_]_0.2% Pt	5.5386	13.7068	364.14	245.9	1.0362	176.2
AgTaO_3__[OMIM][Tf_2_N]_0.2% Pt	5.5673	13.7832	369.97	262.2	1.0342	221.2
AgTaO_3__[TBA][Cl]_0.2% Pt	5.5250	13.6974	362.11	243.8	0.7124	25.1
AgTaO_3__[TPTZ][Cl]_0.2% Pt	5.5345	13.7027	364.84	284.1	0.6986	55.4

**Table 2:** Elemental contents in the surface layer of AgTaO_3_ and AgTaO_3__IL composites doped by Pt. The Pt1, Pt2 and Pt3 fractions of the Pt 4f_7/2_ XPS spectra indicate the relative contribution of platinum species.

-													Pt 4f_7/2_ Fraction (%)		
-	Elements Content (at.%)												Pt1	Pt2	Pt3
Sample Label	Ag	Ta	O	Pt	C	F	B	S	Cl	N	Pt/Ag	C/Ag	Pt-Ag 69.9–70.2 eV	Pt(0), Pt-CO 70.8–71.4 eV	Pt-CxHy, Pt-IL 71.8–72.7 eV
AgTaO_3__0.2%Pt	16.80	22.24	45.56	0.81	14.59	-	-	-	-	-	0.048	0.87	76.25	15.20	8.55
AgTaO_3__[OMIM][BF_4_]_0.2%Pt	15.40	21.96	46.67	0.82	13.10	0.46	0.38	-	-	1.22	0.053	0.85	69.02	22.21	8.77
AgTaO_3__[OMIM][Tf_2_N]_0.2%Pt	16.38	21.96	43.92	0.74	14.93	0.40	-	0.14	-	1.54	0.045	0.91	74.20	17.09	8.71
AgTaO_3__[TBA][Cl]_0.2%Pt	11.61	22.94	43.38	1.20	19.23	-	-	-	0.28	1.36	0.103	1.66	66.77	24.12	9.11
AgTaO_3__[TPTZ][Cl]_0.2%Pt	11.48	23.29	44.50	1.28	16.38	-	-	-	0.35	2.72	0.111	1.43	63.40	27.47	9.13

## Supplementary Materials

The following are available online at https://www.mdpi.com/1996-1944/13/18/4055/s1, Figure S1: FTIR (**a**) and Raman (**b**) spectra of pristine and corresponding ILs and Pt modified AgTaO_3_ samples. Figure S2: The diffusion reflection spectra of the pristine AgTaO_3_ photocatalyst and the corresponding ILs and Pt modified materials. Figure S3: UV-Vis Kubelka–Munk absorption of the pristine AgTaO_3_ photocatalyst and the corresponding ILs and Pt modified materials. Table S1: The analysis average crystallite size and amount H_2_ evolved.
