# Supplementary material for: Fabrication of ILs-Assisted AgTaO3 Nanoparticles for the Water Splitting Reaction: The Effect of ILs on Morphology and Photoactivity
Source: Materials (Basel). 2020 Sep 12;13(18):4055. doi: 10.3390/ma13184055 (PMC7559565; doi:10.3390/ma13184055)
Supplement: Supplementary file 1 [file materials-13-04055-s001.pdf]

materials

Supplementary data

Fabrication of ILs-Assisted $AgTaO_3$ Nanoparticles for the Water Splitting Reaction: The Effect of ILs on Morphology and Photoactivity

Julia Zwara [1], Anna Pancielejko [2], Marta Paszkiewicz-Gawron [1], Justyna Łuczak [2], Magdalena Miodyńska [1], Wojciech Lisowski [3], Adriana Zaleska-Medynska [1] and Ewelina Grabowska-Musiał [1,*]

[1] Department of Environmental Technology, Faculty of Chemistry, University of Gdansk, 80-308 Gdansk, Poland; julia.zwara@phdstud.ug.edu.pl (J.Z.); m.paszkiewicz-gawron@ug.edu.pl (M.P.-G.); magdalena.miodynska@phdstud.ug.edu.pl (M.M.); adriana.zaleska-medynska@ug.edu.pl (A.Z.-M.)
[2] Department of Process Engineering and Chemical Technology, Faculty of Chemistry, Gdansk University of Technology, 80-233 Gdansk, Poland; anna.pancielejko@pg.edu.pl (A.P.); justyna.luczak@pg.edu.pl (J.Ł.)
[3] Institute of Physical Chemistry, Polish Academy of Science, 01-244 Warsaw, Poland; wlisowski@ichf.edu.pl
* Correspondence: ewelina.grabowska@ug.edu.pl; Tel.: +48-58-523-52-22

Table S1. The analysis average crystallite size and amount H_2 evolved.

Sample label	Crystallite size (Å)	Amount of H_2 evolved after 240 min (μmol·g^{-1}) under UV-Vis irradiation
$AgTaO_3$_SS	394	0.14
$AgTaO_3$_HS	288	3.73
$AgTaO_3$_SSR	215	20.4
$AgTaO_3$_SG	373	2.48

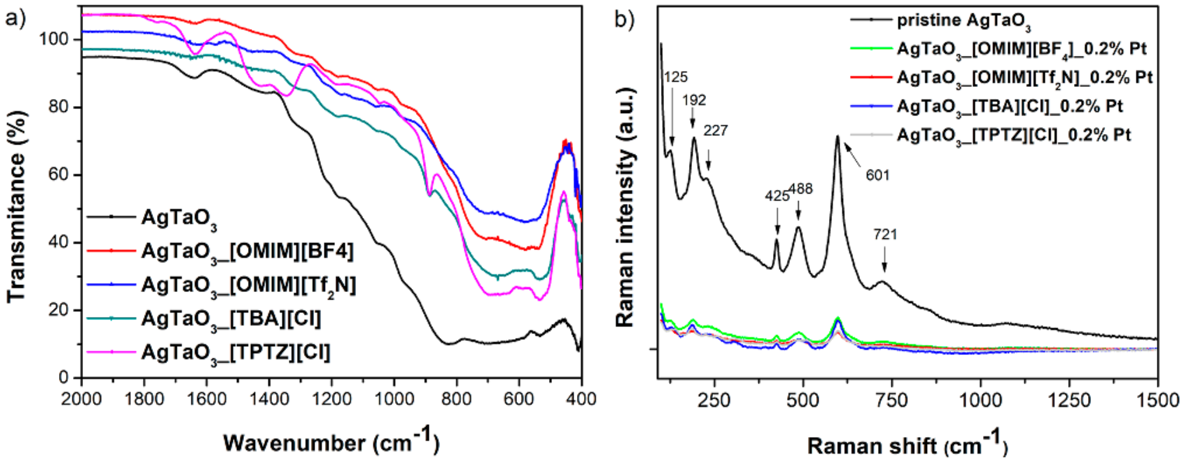

Figure S1. FTIR (**a**) and Raman (**b**) spectra of pristine and corresponding ILs and Pt modified $AgTaO_3$ samples.

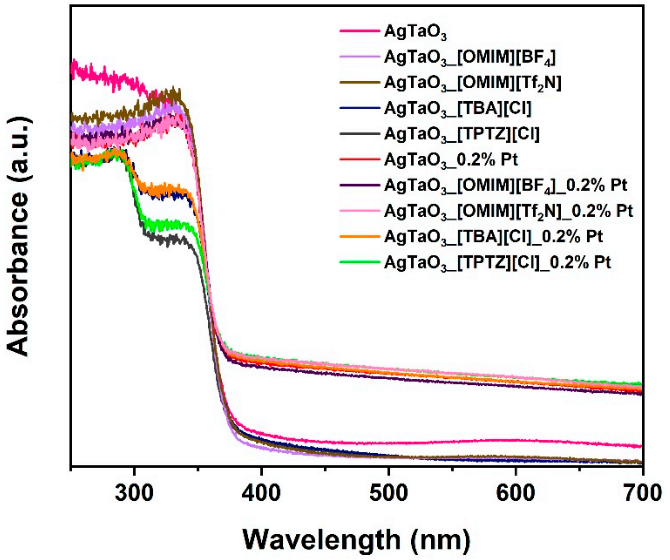

Figure S2. The diffusion reflection spectra of the pristine $AgTaO_3$ photocatalyst and the corresponding ILs and Pt modified materials.

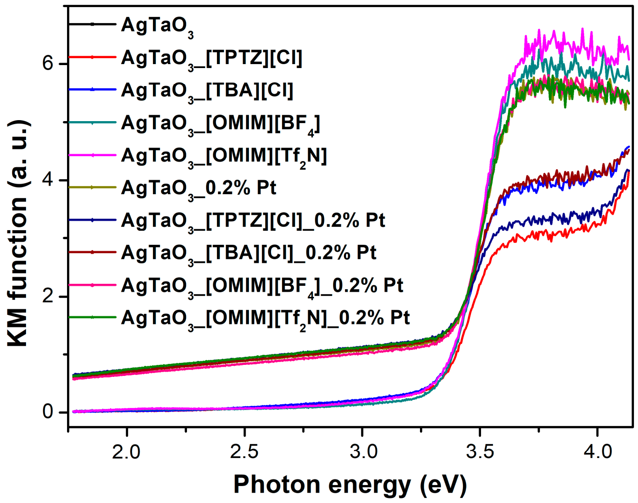

Figure S3. UV-Vis Kubelka–Munk absorption of the pristine $AgTaO_3$ photocatalyst and the corresponding ILs and Pt modified materials.
